# The burden of endometriosis in China from 1990 to 2019

**DOI:** 10.3389/fendo.2022.935931

**Published:** 2022-08-16

**Authors:** Yan Wang, Xiaoyan Wang, Kaijun Liao, Baoqin Luo, Jiashou Luo

**Affiliations:** Department of Gynaecology and Obstetrics, Nanping First Hospital Affiliated to Fujian Medical University, Fujian, China

**Keywords:** Endometriosis, death, disability-adjusted life years, joinpoint regression analysis, age–period–cohort analysis

## Abstract

**Background:**

The trends in deaths from and disability-adjusted life years (DALY) of endometriosis in China remain largely unknown. This study revealed these trends and the effects of age, period, and cohort on the death from and DALY of endometriosis in China from 1990 to 2019.

**Methods:**

Data on endometriosis death and DALY in China between 1990 and 2019 were obtained from the Global Burden of Disease Study 2019 (GBD 2019). The annual percentage change and average annual percent change (AAPC) were analyzed by joinpoint regression. The effects of age, period, and birth cohort on death and DALYs were estimated using an age–period–cohort analysis.

**Results:**

The age-standardized death rate (ASDR) and age-standardized DALY rate for endometriosis significantly decreased in China, with AAPC values of −4.7 (95% confidence interval [CI]: −5.10, −4.30) and −1.2 (95% CI: −1.20, −1.10), respectively. The joinpoint regression analysis showed that the ASDR and age-standardized DALY rate decreased across all age groups. Moreover, the effect of age on endometriosis death and DALY decreased with advancing age. Both the period and cohort effects on endometriosis death and DALY showed decreasing trends, with the effects on death decreasing faster than the effects on DALY.

**Conclusions:**

The endometriosis ASDR and age-standardized DALY rate decreased from 1990 to 2019. The effects of the period and birth cohort on endometriosis death and DALY showed a declining trend across all age groups. The effect of age on endometriosis deaths and DALYs decreased with advancing age.

## 1 Introduction

Endometriosis is an estrogen-dependent chronic gynecological disorder in which tissue resembling the endometrium develops outside the uterus, typically in the pelvic area (1). Globally, approximately 190 million women suffer from endometriosis, accounting for 10%–15% of women of reproductive age (2). Almost half of the women with endometriosis experience painful intercourse, which disrupts normal sexual function (3). Additionally, endometriosis is commonly concomitant with depression and anxiety (4).

Endometriosis is one of the most common and difficult clinical diseases in gynecology. It mostly occurs among women of childbearing age. The incidence rate has increased significantly recently (5). The disease is one of the main causes of pelvic pain and infertility in women of childbearing age, which seriously affects the women’s quality of life (2). Although endometriosis is a benign disease, it has malignant behaviors such as invasion, implantation, recurrence, etc., also known as “good cancer” (6). Therefore, the prevention of this disease is far more important than the treatment. Recently, due to the popularization of laparoscopy, the incidence of endometriosis, especially in mild patients, has increased significantly in China (7).

Traditionally, epidemiological studies have focused on the incidence and prevalence of endometriosis, and few studies have focused on death and disability-adjusted life years (DALY) (8–10). The DALY is a widely-used gap measure representing the sum of years of life lost (YLL) due to years lived with disability (YLD) and premature mortality, and is an essential indicator of the burden of disease. The Global Burden of Diseases, Injuries, and Risk Factors Study (GBD) annually updates its data, often changing the methods to make the data more comprehensive and appropriate. Therefore, the latest data on endometriosis can be obtained from the GBD 2019 (11). To the best of our knowledge, this is the first study on endometriosis death and DALY that is based on data from the GDB 2019.

In this study, joinpoint and age–period–cohort analyses were used to estimate the trends in death and DALY and to explore the effects of age, period, and birth cohort on endometriosis in China from 1990 to 2019, based on data obtained from the GBD 2019. Our findings are expected to provide useful information for further investigation of this disease.

## 2 Materials and methods

### 2.1 Data sources

Endometriosis death and DALY were obtained from the GBD 2019, which provides a comprehensive assessment of the burden of 369 diseases (e.g., incidence, mortality, and DALY) with 87 risk factors and from 204 countries and territories (http://ghdx.healthdata.org/gbd-results-tool) between 1990 and 2019 (11, 12). Endometriosis deaths and DALYs were age-standardized based on the GBD 2019 global age-standard population. To estimate the effects of age, period, and birth cohort, the age-standardized death rate (ASDR) and age-standardized DALY rate were recorded into consecutive 5-year age groups (15–19, 20–24, …, 45–49, 50–54), continuous 5-year periods from 1990–1994 to 2015–2019, and corresponding successive 5-year birth cohorts from 1938–1942 to 1998–2002. This study was approved by the Nanping First Hospital Affiliated to Fujian Medical University.

### 2.2 Statistical analysis

#### 2.2.1 Joinpoint regression analysis

A joinpoint regression analysis was performed to describe the trends in death and DALY. The results were expressed as the annual percentage change (APC), average annual percent change (AAPC), and 95% confidence intervals (CIs) for each age, period, and cohort group. Analysis was performed using the Joinpoint Regression Program v4.6.0.0 (April 2018), developed by the Surveillance Research Program of the U.S. National Cancer Institute.

#### 2.2.2 Age–period–cohort analysis

An age–period–cohort analysis was conducted to reflect the different effects of age, period, and cohort on the incidence and mortality trends of various diseases (13–15). However, this analysis failed to estimate the specific effects of age, period, and cohort because of the collinear relationships between these three variables (i.e., cohort = period − age). A feasible intrinsic estimator (IE) method was developed by Yang and Fu in 2008 to distinguish these three effects (16). In this study, an age–period–cohort analysis using the IE method was conducted to estimate the effects of age, period, and cohort on the death from and DALY of endometriosis. Groups encompassing individuals aged <15 years or >55 years were excluded. The APC model is based on a log-linear model and can be written as follows (17):

Y = log(M) = µ + α(age)i + β(period)j + γ(cohort)k + ϵ

where M denotes the incidence of endometriosis, µ and ϵ are the intercept and random error, and α(age)i, β(period)j, and γ(cohort)k denote the effects of age group α, time period β, and birth cohort k, respectively. The goodness of fit for each model was estimated using the likelihood ratio test and Akaike information criterion. P-value <0.05 was considered to indicate statistical significance. Standard error coefficients and risk ratios (RRs) were also calculated. The relative risk was set as the exponential value of the coefficient. The age–period–cohort analysis was performed using Stata software (v15.0; Stata Corp, College Station, TX, USA).

Joinpoint Regression analysis, also known as the piecewise regression model, is a statistical method to analyze the trend of disease over time (18). The basic idea of joinpoint regression model analysis is to divide a long-term trend line into several statistically significant trend segments through model fitting, and each segment is described by a continuous linear line. The connection points between different trend segments are called turning points. The number of turning points and statistical significance tests was judged using the Monte Carlo permutation method (18).

## 3 Results

### 3.1 Descriptive analysis of endometriosis death and DALY rates in China

The trends of death and DALY for endometriosis across all ages from 1990 to 2019 are presented in **Table 1**. Generally, the ASDR and age-standardized DALY rate decreased from 1990 to 2019, with AAPC values of −4.7 (95% CI: −5.1, −4.3) and −1.2 (95% CI: −1.2, −1.1), respectively. The ASDR sharply declined from 1990 to 1996, and then slightly increased from 1996 to 2004. However, it dramatically decreased again from 2004 to 2019 with an APC of −8.2 (95% CI: −8.5, −7.9). The age-standardized DALY rate showed a declining trend, barring the period from 2010 to 2017, with a mild upward trend (APC = 0.4, 95% CI: 0.3, 0.5). The age-standardized DALY rate declined the most between 2005 and 2010, with an APC of −4.3 (95% CI: −4.4, −4.1).

**Table 1:** Trends in endometriosis death and DALY rates in China, 1990–2019.

Segments	Year	APC * (95% CI)
ASDR		
trend1	1990–1996	−4.1* (−5.50, −2.70)
trend2	1996–2004	1.9* (0.70, 3.00)
trend3	2004–2019	−8.2* (−8.50, −7.90)
AAPC*	1990–2019	−4.7* (−5.10, −4.30)
**Age-standardized DALY rate**		
trend1	1990–1994	−1.6* (−1.80, −1.40)
trend2	1994–2005	−0.2* (−0.30, −0.20)
trend3	2005–2010	−4.3* (−4.40, −4.10)
trend4	2010–2017	0.4* (0.30, 0.50)
trend5	2017–2019	−2.8* (−3.30, −2.20)
AAPC*	1990–2019	−1.2* (−1.20, −1.10)

APC, annual percentage change; AAPC, average annual percent change; CI, confidence interval; ASDR, age−standardized death rate; DALY, disability-adjusted life years; *Indicates statistical significance <0.05.

### 3.2 Joinpoint regression analysis

The AAPCs and APCs in the death and DALY of endometriosis in China from 1990 to 2019 are shown in **Table 2**. The age-specific rates showed decreasing trends in death and DALY. In comparison, the ASDR significantly decreased, with AAPC values fluctuating between −6.4 and −3.7 for all age groups, while the age-standardized DALY rate showed a relatively slight decline, with AAPC values of −1.3 to −0.9. Overall, deaths and DALYs of endometriosis decreased for all age groups in China from 1990 to 2019.

**Table 2:** The average annual percent changes (AAPCs) in death and DALY of endometriosis in China, 1990–2019.

Age Group	Death (AAPC)	DALY(AAPC)
Age-standardized rate	−4.7* (−5.10, −4.30)	−1.2* (−1.20, −1.10)
15–19	−6.4* (−6.8, −6.0)	−0.9* (−1.0, −0.8)
20–24	−5.7* (−6.3, −5.1)	−1.0* (−1.1, −0.9)
25–29	−5.6* (−6.7, −4.5)	−1.1* (−1.2, −1.1)
30–34	−5.8* (−6.7, −4.8)	−1.2* (−1.3, −1.2)
35–39	−5.2* (−5.8, −4.7)	−1.3* (−1.3, −1.2)
40–44	−4.9* (−5.4, −4.4)	−1.2* (−1.2, −1.1)
45–49	−4.7* (−5.3, −4.0)	−1.1* (−1.1, −1.0)
50–54	−3.7* (−4.3, −3.2)	−1.0* (−1.1, −1.0)

### 3.3 Age–period–cohort effects

**Figure 1:**
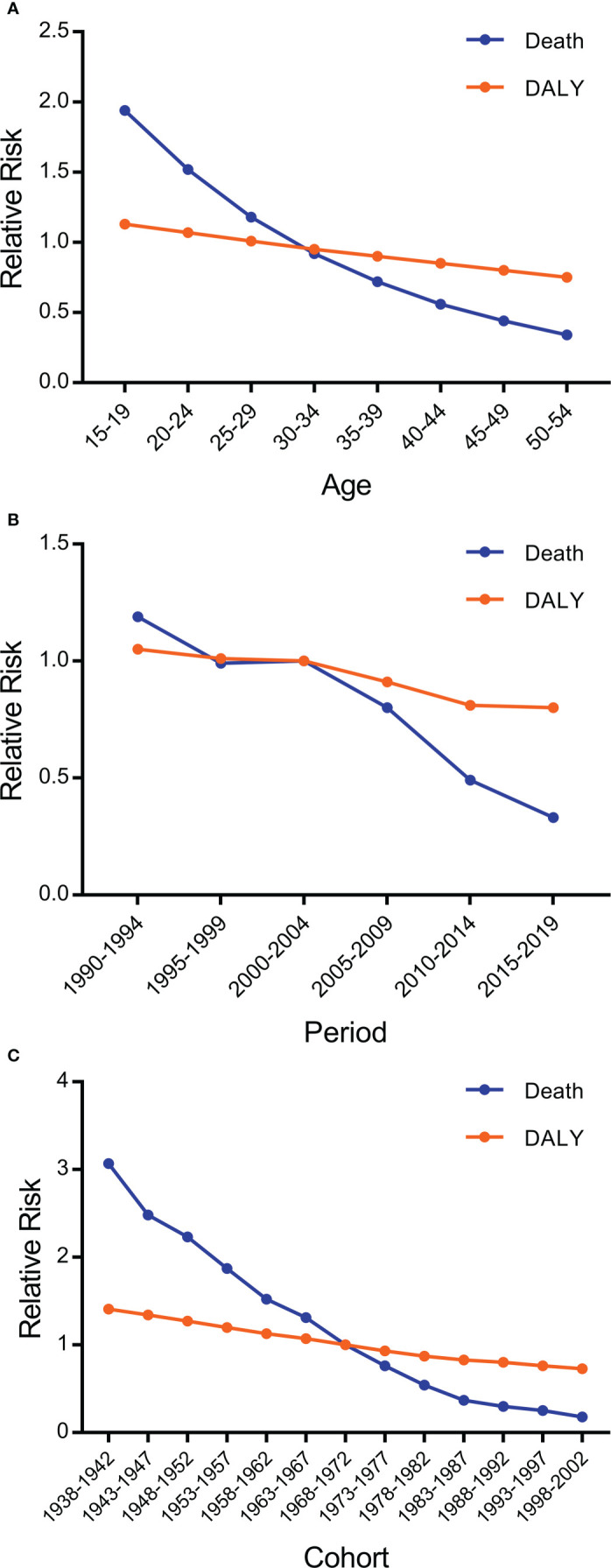
Death and disability-adjusted life years (DALY) relative risks of endometriosis due to **(A)** age, **(B)** period, and **(C)** cohort effects.

The age–period–cohort model was used to estimate the effects of age, period, and cohort on death and DALY of endometriosis. The coefficients and estimated RRs of age, period, and cohort are displayed in **Figure 1**; **Tables 2**, **3**.

**Table 3:** Age−period−cohort model analysis results of endometriosis death and DALY and relative risks (RR) in China.

Factor	Death (RR, 95% CI)	DALY (RR, 95% CI)
**Age**		
15–19	1.94 (0.37, 10.16)	1.13 (1.12, 1.15)
20–24	1.52 (0.36, 6.38)	1.07 (1.06, 1.08)
25–29	1.18 (0.34, 4.13)	1.01 (1, 1.02)
30-34	0.92 (0.31, 2.8)	0.95 (0.94, 0.96)
35–39	0.72 (0.26, 2.03)	0.9 (0.89, 0.91)
40–44	0.56 (0.2, 1.59)	0.85 (0.84, 0.86)
45–49	0.44 (0.14, 1.35)	0.8 (0.79, 0.81)
50–54	0.34 (0.1, 1.22)	0.75 (0.74, 0.76)
**Period**		
1990–1994	1.19 (0.38, 3.74)	1.05 (1.03, 1.06)
1995–1999	0.99 (0.37, 2.68)	1.01 (1.00, 1.02)
2000–2004	1.00 (1.00, 1.00)	1.00 (1.00, 1.00)
2005–2009	0.80 (0.30, 2.09)	0.91 (0.90, 0.92)
2010–2014	0.49 (0.16, 1.54)	0.81 (0.80, 0.82)
2015–2019	0.33 (0.09, 1.23)	0.80 (0.80, 0.81)
**Cohort**		
1938–1942	3.07 (0.31, 30.72)	1.41 (1.35, 1.48)
1943–1947	2.48 (0.51, 12.20)	1.34 (1.31, 1.38)
1948–1952	2.23 (0.62, 8.07)	1.27 (1.25, 1.29)
1953–1957	1.87 (0.57, 6.09)	1.20 (1.19, 1.22)
1958–1962	1.52 (0.48, 4.77)	1.13 (1.12, 1.15)
1963–1967	1.31 (0.44, 3.87)	1.07 (1.06, 1.08)
1968–1972	1.00 (1.00, 1.00)	1.00 (1.00, 1.00)
1973–1977	0.76 (0.18, 3.12)	0.93 (0.92, 0.94)
1978–1982	0.54 (0.08, 3.70)	0.87 (0.86, 0.88)
1983–1987	0.37 (0.03, 4.09)	0.83 (0.82, 0.84)
1988–1992	0.30 (0.01, 8.22)	0.80 (0.79, 0.81)
1993–1997	0.25 (0.00, 32.81)	0.76 (0.74, 0.79)
1998–2002	0.18 (0.00, 2,149.22)	0.73 (0.68, 0.79)

#### 3.3.1 Effect of age

The RRs and coefficients of the effect of age on endometriosis death and DALY in China are shown in **Figure 1A**; **Tables 3**, **4**. After controlling for the effects of period and cohort, the effect of age on death and DALY decreased with advancing age. For death, the RR decreased by 82.5% from 15–19 years to 50–54 years. For DALY, compared with 15–19 years, the RR of 50–54 years decreased by 33.6%. In summary, the effects of age on death and DALY of endometriosis displayed a trend of decreasing with advancing age.

**Table 4:** Age–period–cohort (APC) model analysis results of endometriosis death and DALY rates in China.

Variable	Death (Coef, 95% CI)	DALY (Coef, 95% CI)
**Age**		
15–19	0.66 (−0.99, 2.32)	0.13 (0.11, 0.14)
20–24	0.42 (−1.02, 1.85)	0.07 (0.06, 0.08)
25–29	0.17 (−1.08, 1.42)	0.01 (0, 0.02)
30–34	−0.08 (−1.19, 1.03)	−0.05 (−0.06, −0.04)
35–39	−0.33 (−1.36, 0.71)	−0.11 (−0.12, −0.1)
40–44	−0.57 (−1.61, 0.46)	−0.17 (−0.18, −0.16)
45–49	−0.82 (−1.94, 0.3)	−0.23 (−0.24, −0.21)
50–54	−1.07 (−2.34, 0.2)	−0.28 (−0.3, −0.27)
**Period**		
1990–1994	0.17 (−0.98, 1.32)	0.04 (0.03, 0.06)
1995–1999	−0.01 (−1, 0.99)	0.01 (0, 0.02)
2000–2004	0 (0, 0)	0 (0, 0)
2005–2009	−0.22 (−1.19, 0.74)	−0.09 (−0.1, −0.08)
2010–2014	−0.71 (−1.85, 0.43)	−0.21 (−0.22, −0.2)
2015–2019	−1.1 (−2.41, 0.21)	−0.22 (−0.23, −0.21)
**Cohort**		
1938–1942	1.12 (−1.18, 3.42)	0.35 (0.3, 0.39)
1943–1947	0.91 (−0.68, 2.5)	0.29 (0.27, 0.32)
1948–1952	0.8 (−0.48, 2.09)	0.24 (0.22, 0.26)
1953–1957	0.62 (−0.56, 1.81)	0.19 (0.17, 0.2)
1958–1962	0.42(−0.73, 1.56)	0.13 (0.11, 0.14)
1963–1967	0.27 (−0.82, 1.35)	0.07 (0.05, 0.08)
1968–1972	0 (0, 0)	0 (0, 0)
1973–1977	−0.28 (−1.69, 1.14)	−0.07 (-0.08, −0.06)
1978–1982	−0.62 (−2.54, 1.31)	−0.14 (-0.15, −0.12)
1983–1987	−0.98 (−3.37, 1.41)	−0.18 (−0.2, −0.17)
1988–1992	−1.21 (−4.52, 2.11)	−0.22 (−0.24, −0.21)
1993–1997	−1.41 (−6.32, 3.49)	−0.27 (−0.3, −0.24)
1998–2002	−1.69 (−∞, 7.67)	−0.31 (−0.38, −0.23)

#### 3.3.2 Effect of period

The effects of the period showed decreasing trends for death and DALY (**Figure 1B**; **Tables 3**, **4**). The RR of death decreased significantly, but that of DALY decreased only mildly. From 1992 to 2017, the RR of death decreased by 72.3% and that of DALY decreased by 23.8%. The RR of death decreased relatively faster after 2002, whereas the RR of DALY was relatively more stable. Overall, the effects of the period on death and DALY revealed that the RRs of death and DALY of endometriosis decreased with time.

#### 3.3.3 Effect of period cohort

The effect of the cohort displayed downward trends for both death and DALY (**Figure 1C**; **Table 2**). Overall, the RR of death decreased faster than that of DALY. From the birth cohorts of 1938–1942 to 1998–2002, the RRs of death and DALY decreased significantly, by 94.1% and 48.2%, respectively.

## 4 Discussion

This study investigated the long-term trends in the risk of death and DALY for endometriosis among women in China from 1990 to 2019. It was one of the first investigations of the effects of age, period, and cohort on endometriosis death and DALY in China to use the IE method of age–period–cohort analysis. Findings from this study will provide a factual basis for preventing endometriosis in the future.

Overall, both the ASDR and age-standardized DALY rate decreased between 1990 and 2019 in China. However, the DALY rate decreased at a slower rate than the ASDR. The ASDR showed an upward trend during 1996–2004, with an APC value of 1.9 (95% CI: 0.70, 3.00). The DALY also showed a slight upward trend in 2010–2017, with an APC value of 0.4 (95% CI: 0.30, 0.50). The ASDR and DALY showed the same downward trend for all age groups. Compared with the ASDR, the DALY across all age groups changed in a less drastic manner.

Endometriosis is a common and frequently occurring disease in women of reproductive age. Age reflects the influence of physiological factors on reproductive ability. The risk of death decreased significantly from 15–19 years to 50–54 years, while that of DALY declined relatively mildly. The death and DALY of endometriosis are mainly affected by its prevalence, and the trend of age-specific prevalence of endometriosis in China (unpublished data) is consistent with the trend of death and DALY of endometriosis recorded in this study, which overall decreased with advancing age. This trend might be associated with increased urbanization, improved medical technology, and increased health awareness among women.

The effects of periods often reflected the impacts of specific time periods on diseases, death, and DALY across all age groups, mainly mediated by social, economic, and medical factors. The RR of the period on endometriosis death and DALY revealed that the risk of developing endometriosis significantly decreased from 1992 to 2017. In contrast, the RR of DALY decreased more rapidly than that of death. Several previous studies have demonstrated that while the death rate of endometriosis is very low, patients almost always experience pain (19–21). Unfortunately, the complete eradication of endometriosis-associated pain can rarely be achieved. Therefore, although the medical condition appeared to improve, the RR of DALY decreased more slowly than that of death.

The birth cohort effects reflect the diverse risk factors that affect different birth cohorts in early life, such as environmental, behavioral, and socioeconomic factors. The effect of birth cohort on DALY for endometriosis in China decreased slowly from the 1938–1942 cohort to the 1998–2002 cohort, whereas the effect on death decreased more rapidly. This trend probably arose because, compared with earlier birth cohorts, the more recent birth cohorts had a stronger awareness of disease prevention and received a better education. The reliable diagnosis of endometriosis currently requires surgical visualization, which is most commonly done *via* laparoscopy (22, 23). Over the past few decades, both diagnosis and treatment have improved because of improvements in laparoscopic techniques. Additionally, the Healthy China 2030 program aims to promote health care in China and potentially reduce the risk of developing endometriosis (24).

Improvements in the medical environment and technology are also potential reasons for the decline in endometriosis mortality, and studies have shown that increased medical insurance coverage can reduce endometriosis mortality (25). Recently, the health service input indicators for China, such as the number of health institutions, the number of health personnel, the total building area, and financial subsidy income, have all increased at different rates (26). The convenience and fairness of access to health services allow our Chinese residents to obtain more and better health services (27). The improvement of the medical insurance system and the increase in the coverage rate have resulted in more Chinese residents receiving medical services, increasing the early diagnosis and treatment of diseases such as endometriosis, and thus reducing their disease burden to a certain extent (28).

### 4.1 Limitations

This study not only provided the most updated evaluation of the trends in death and DALYs of endometriosis in China but also elucidated the effects of age, cohort, and period by age–period–cohort analysis. Meanwhile, some limitations of our work should also be identified. Firstly, age–period–cohort analysis with the IE method was an ecological study, for which we were unable to make casual inference. We can only make some assumptions based on the present data and the literature. Therefore, further research on the contributing factors should be conducted. Second, owing to a lack of relevant data, differences in the trends of death and DALY of endometriosis between urban and rural China could not be determined. Thus, the changes in trends between urban and rural China remain to be investigated in further studies. Despite its limitations, this study is nationally significant and was the first of its kind to use age–period–cohort modeling to explore trends in the death and DALY of endometriosis in China.

## 5 Conclusions

The ASDR and age-standardized DALY rates of endometriosis showed a decreasing trend from 1990 to 2019. Age was the most significant factor in risk determination. The effect of age on death and DALY displayed a downward trend with advancing age. Both the effects of period and cohort on death and DALY exhibited downward trends.

## Data availability statement

The datasets presented in this study can be found in online repositories. The names of the repository/repositories and accession number(s) can be found below: http://ghdx.healthdata.org/gbd-results-tool.

## Author contributions

JL conceived the study. YW, XW, and KL collected and analyzed the data. BL interpreted the results. YW wrote the first draft of the manuscript. JL and XW revised and finalized the manuscript. All authors listed have made a substantial, direct, and intellectual contribution to the work and approved it for publication.

## Funding

This research was funded by the Startup Fund for scientific research, Fujian Medical University (Grant number: 2019QH1247).

## Conflict of interest

The authors declare that the research was conducted in the absence of any commercial or financial relationships that could be construed as a potential conflict of interest.

## Publisher’s note

All claims expressed in this article are solely those of the authors and do not necessarily represent those of their affiliated organizations, or those of the publisher, the editors and the reviewers. Any product that may be evaluated in this article, or claim that may be made by its manufacturer, is not guaranteed or endorsed by the publisher.

## References

[1] Zondervan KT Becker CM Missmer SA. Endometriosis. N Engl J Med (2020) 382(13):1244–56. doi: 10.1056/NEJMra1810764 32212520

[2] Shafrir AL Farland LV Shah DK Harris HR Kvaskoff M Zondervan K. Risk for and consequences of endometriosis: A critical epidemiologic review. Best Pract Res Clin Obstet Gynaecol (2018) 51:1–15. doi: 10.1016/j.bpobgyn.2018.06.001 30017581

[3] Pluchino N Wenger JM Petignat P Tal R Bolmont M Taylor HS. Sexual function in endometriosis patients and their partners: Effect of the disease and consequences of treatment. Hum Reprod Update (2016) 22(6):762–74. doi: 10.1093/humupd/dmw031 27591248

[4] Friedl F Riedl D Fessler S Wildt L Walter M Richter R. Impact of endometriosis on quality of life, anxiety, and depression: an Austrian perspective. Arch Gynecol Obstet (2015) 292(6):1393–9. doi: 10.1007/s00404-015-3789-8 26112356

[5] Taylor HS Kotlyar AM Flores VA. Endometriosis is a chronic systemic disease: clinical challenges and novel innovations. Lancet (2021) 397(10276):839–52. doi: 10.1016/s0140-6736(21)00389-5 33640070

[6] Dai Y Li X Shi J Leng J. A review of the risk factors, genetics and treatment of endometriosis in Chinese women: A comparative update. Reprod Health (2018) 15(1):82. doi: 10.1186/s12978-018-0506-7 29783992 PMC5963030

[7] Agarwal SK Chapron C Giudice LC Laufer MR Leyland N Missmer SA. Clinical diagnosis of endometriosis: a call to action. Am J Obstet Gynecol (2019) 220(4):354.e351–354.e312. doi: 10.1016/j.ajog.2018.12.039 30625295

[8] Fuldeore MJ Soliman AM. Prevalence and symptomatic burden of diagnosed endometriosis in the united states: National estimates from a cross-sectional survey of 59,411 women. Gynecol Obstet Invest (2017) 82(5):453–61. doi: 10.1159/000452660 27820938

[9] Soliman AM Singh S Rahal Y Robert C Defoy I Nisbet P. Cross-sectional survey of the impact of endometriosis symptoms on health-related quality of life in Canadian women. J Obstet Gynaecol Can (2020) 42(11):1330–8. doi: 10.1016/j.jogc.2020.04.013 32758398

[10] Singh S Soliman AM Rahal Y Robert C Defoy I Nisbet P. Prevalence, symptomatic burden, and diagnosis of endometriosis in Canada: Cross-sectional survey of 30 000 women. J Obstet Gynaecol Can (2020) 42(7):829–38. doi: 10.1016/j.jogc.2019.10.038 32001176

[11] Global burden of 369 diseases and injuries in 204 countries and territories, 1990-2019: a systematic analysis for the global burden of disease study 2019. Lancet (2020) 396(10258):1204–22. doi: 10.1016/s0140-6736(20)30925-9 PMC7567026 33069326

[12] Global burden of 87 risk factors in 204 countries and territories, 1990-2019: A systematic analysis for the global burden of disease study 2019. Lancet (2020) 396(10258):1223–49. doi: 10.1016/s0140-6736(20)30752-2 PMC7566194 33069327

[13] Lee HY Kim DK Doo SW Yang WJ Song YS Lee B. Time trends for prostate cancer incidence from 2003 to 2013 in south Korea: An age-Period-Cohort analysis. Cancer Res Treat (2020) 52(1):301–8. doi: 10.4143/crt.2019.194 PMC6962480 31401823

[14] Liu X Zhou M Yu C Zhang ZJ. Age-Period-Cohort analysis of type 2 diabetes mortality attributable to particulate matter pollution in China and the U. S J Diabetes Res (2020) 2020:1243947. doi: 10.1155/2020/1243947 32626775 PMC7306083

[15] Okui T. Age-period-cohort analysis of cardiovascular disease mortality in Japan, 1995-2018. J Prev Med Public Health (2020) 53(3):198–204. doi: 10.3961/jpmph.20.037 32498145 PMC7280805

[16] Yang Y Schulhofer-Wohl S Fu WJJ Land KC. The intrinsic estimator for age-period-cohort analysis: What it is and how to use it. Am J Sociology (2008) 113(6):1697–736. doi: 10.1086/587154

[17] Tzeng IS Chen KH Lee YL Yang WS. Trends and age-Period-Cohort effects of fertility rate: Analysis of 26,224 married women in Taiwan. Int J Environ Res Public Health (2019) 16(24):4952. doi: 10.3390/ijerph16244952 PMC6950473 31817631

[18] Qiao YL Taylor PR Yao SX Schatzkin A Mao BL Lubin J. Relation of radon exposure and tobacco use to lung cancer among tin miners in yunnan province, China. Am J Ind Med (1989) 16(5):511–21. doi: 10.1002/ajim.4700160504 2589328

[19] Maddern J Grundy L Castro J Brierley SM. Pain in endometriosis. Front Cell Neurosci (2020) 14:590823. doi: 10.3389/fncel.2020.590823 33132854 PMC7573391

[20] Wei Y Liang Y Lin H Dai Y Yao S. Autonomic nervous system and inflammation interaction in endometriosis-associated pain. J Neuroinflamm (2020) 17(1):80. doi: 10.1186/s12974-020-01752-1 PMC7060607 32145751

[21] Kim JH Han E. Endometriosis and female pelvic pain. Semin Reprod Med (2018) 36(2):143–51. doi: 10.1055/s-0038-1676103 30566980

[22] Tavcar J Loring M Movilla PR Clark NV. Diagnosing endometriosis before laparoscopy: Radiologic tools to evaluate the disease. Curr Opin Obstet Gynecol (2020) 32(4):292–7. doi: 10.1097/gco.0000000000000638 32398583

[23] Han XT Guo HY Kong DL Han JS Zhang LF. Analysis of characteristics and influence factors of diagnostic delay of endometriosis. Zhonghua Fu Chan Ke Za Zhi (2018) 53(2):92–8. doi: 10.3760/cma.j.issn.0529-567X.2018.02.005 29534377

[24] Tan X Zhang Y Shao H. Healthy China 2030, a breakthrough for improving health. Glob Health Promot (2019) 26(4):96–9. doi: 10.1177/1757975917743533 29297762

[25] Vercellini P Viganò P Somigliana E Fedele L. Endometriosis: pathogenesis and treatment. Nat Rev Endocrinol (2014) 10(5):261–75. doi: 10.1038/nrendo.2013.255 24366116

[26] Deng F Lv JH Wang HL Gao JM Zhou ZL. Expanding public health in China: an empirical analysis of healthcare inputs and outputs. Public Health (2017) 142:73–84. doi: 10.1016/j.puhe.2016.10.007 28057203

[27] Li H Liu K Gu J Zhang Y Qiao Y Sun X. The development and impact of primary health care in China from 1949 to 2015: A focused review. Int J Health Plann Manage (2017) 32(3):339–50. doi: 10.1002/hpm.2435 28670754

[28] Dai Y Zhang JJ Lang JH Zhou YF Guo HY Zhang XM. A convenience sampling questionnaire survey of the current status of diagnosis and treatment of endometriosis in China in 2018. Zhonghua Fu Chan Ke Za Zhi (2020) 55(6):402–7. doi: 10.3760/cma.j.cn112141-20191213-00669 32842247

